# Polypharmacy in older patients: identifying the need for support by a community pharmacist

**DOI:** 10.1186/s12877-019-1276-y

**Published:** 2019-10-21

**Authors:** Jean-Baptiste Beuscart, Ségolène Petit, Sophie Gautier, Patrick Wierre, Thibaut Balcaen, Jean-Marc Lefebvre, Nicolas Kambia, Elisabeth Bertoux, Daniel Mascaut, Christine Barthélémy, Damien Cuny, François Puisieux, Bertrand Décaudin

**Affiliations:** 1 University of Lille, EA 2694 - Santé publique: épidémiologie et qualité des soins, F-59000 Lille, France; 2 Department of Geriatrics, CHU Lille, F-59000 Lille, France; 3 Department of Pharmacy, CHU Lille, F-59000 Lille, France; 4 Department of Pharmacology, CHU Lille, F-59000 Lille, France; 5 Association des Conseillers et des Pharmaciens Agréés Maîtres de stage du Nord Pas de Calais, Lille, France; 6 Department of Public Health, CHU Lille, F-59000 Lille, France; 7 Department of General Practice, University of Lille, F-50045 Lille Cedex, France; 8 University of Lille, EA 7365 - Groupe de recherche sur les injectables et les technologies associées, F-59000 Lille, France; 9 University of Lille, EA 4483, Impacts de l’environnement chimique sur la santé humaine (IMPECS), F- 59000 Lille, France

## Abstract

**Background:**

The community pharmacist is a key player in medication reviews of older outpatients. However, it is not always clear which individuals require a medication review. The objective of the present study was to identify high-priority older patients for intervention by a community pharmacist.

**Methods:**

As part of their final-year placement in a community pharmacy, pharmacy students conducted 10 interviews each with older adults (aged 65 or over) taking at least five medications daily. The student interviewer also offered to examine the patient’s home medicine cabinet. An interview guide was developed by an expert group to assess the difficulties in managing and taking medications encountered by older patients.

**Results:**

The 141 students interviewed a total of 1370 patients (mean age: 81.5; mean number of medications taken daily: 9.3). Of the 1370 interviews, 743 (54.2%) were performed in the patient’s home, and thus also included an examination of the home medicine cabinet. Adverse events were reported by 566 (42.0%) patients. A total of 378 patients (27.6%) reported difficulties in preparing, administering and/or swallowing medications. The inspections of medicine cabinets identified a variety of shortcomings: poorly located cabinets (in 15.0% of inspections), medication storage problems (21.7%), expired medications (40.7%), potentially inappropriate medications (15.0%), several different generic versions of the same drug (19.9%), and redundant medications (20.4%).

**Conclusions:**

In a community pharmacy setting, high-priority older patients for intervention by a community pharmacist can be identified by asking simple questions about difficulties in managing, administering, taking or storing medications.

## Background

Polypharmacy exposes older adults to an increased risk of adverse drug reactions [1–3], and has a significant impact on mortality and the likelihood of hospitalization [4, 5]. Several interventions aimed at reducing this risk have been suggested, with a notable focus on detecting and reducing potentially inappropriate prescriptions [6]. These interventions require healthcare professionals to be more aware of at-risk situations and patients requiring particular assistance. In France, the community pharmacist now has an increasingly important and changing role in care provision for patients with chronic diseases, and can even become a patient’s designated pharmacist. This status enables the pharmacist to adapt the patient’s treatment (in collaboration with the patient’s family physician), notably on the basis of a medication review.

The Pharmaceutical Care Network Europe working group on medication review has defined the latter as “*a structured evaluation of a patient’s medicines with the aim of optimizing medicines use and improving health outcomes. This entails detecting drug related problems and recommending interventions*”. A formal medication review includes several components: an assessment of treatment adherence and safety, the identification of any drug interactions, reminders about good administration practice, appropriate medication use, and feedback to the prescribing physician(s). Medication review is known to be associated with a decrease in the number of drug related problems and inappropriate prescription [7, 8]. However, the other putative benefits of medication review’s (in terms of less frequent hospitalizations and reduced mortality) are subject to debate [9, 10]. Moreover, it is not always clear which individuals require a medication review. Several studies were conducted with the purpose of better identifying patients at risk of adverse drug events but their predictive value was low and few of them were conducted among older patients [11]. One can hardly determine which older patient should be prioritized for medication review.

Improved cross-disciplinary communication increases the success rate for this type of intervention [12, 13] but requires community pharmacists or pharmacy students to be trained accordingly [14, 15]. Faculties of pharmacy are now focusing on training students to perform these new duties. Specific teaching units are based on real-life scenarios and their application during an internship – typically the community pharmacy internship for final-year students. At the Lille Faculty of Pharmacy, the final-year internship always includes work related to the patient’s care pathway [16]. In 2015, this work addressed good prescribing practice for older patients with polypharmacy.

The primary objective of the present study was to identify high-priority patients for medication review in a community pharmacy setting. To this end, we assessed the difficulties in managing and taking medications encountered by older patients.

## Methods

### Study design

This was a cross-sectional study performed between January 5th and June 30th, 2015, in community pharmacies in the Nord-Pas-de-Calais region of France. Each of the 141 sixth-year student interns was asked to interview 10 older patients (aged 65 and over) taking at least five medications daily, in order to assess their home medication management. This interview could be performed in the pharmacy or (if the patient agreed) at the patient’s home. In the latter case, the interviewer examined the patient’s home medicine cabinet and assessed medication storage.

### Ethical aspects

The need for consent was waived by the local independent ethics committee (CPP Nord-Ouest IV, Lille, France), which decided that the study was non-interventional. People could oppose the collection of their data at the time of the interview or could ask the responsible of the data (Pr B. Decaudin) to remove their data from the data base at any time after the interview. Consequently, in line with French legislation, a formal approval was not required but there was a formal information, which gave the right to the people to access, modify or remove their data upon request. The study was registered with the French National Data Protection Commission (CNIL, Paris, France; reference: 1826665).

### Study preparation

A working group (comprising faculty members, community pharmacists, family physicians, and geriatricians) drew up several standardized study documents: (i) an interview guide, (ii) guidance on interviewing and collecting information, and (iii) a letter for the patient’s family physician. Moreover, community pharmacists in eight towns in the region attended seminars on good practice in drug use in older patients.

### Interviews and data collection

The pharmacy students presented the project to the community pharmacy’s staff, detected potential interviewees, sent a letter to the patient’s family physician, made an appointment (in agreement with the patient and the supervising community pharmacist), and prepared and conducted the interview. Convenience sampling was used. If a patient was not able to come to the pharmacy, the patient’s primary carer was invited for the interview. The data on the patient’s medications were collected in a three-section interview grid: (i) information on the patient and his/her medications; (ii) the patient and/or carer’s level of knowledge about the medications, and the level of adherence (on the questionnaire recommended by the French national health insurance [17]); and (iii) management of medications, with questions on whether the patient could prepare and/or administer medications on his/her own, and possible difficulties in preparing and administering medications. The questionnaire is provided in Additional file 1. Geriatric syndromes were assessed by simple questions. For each medicine, the patient’s knowledge about indication was investigated. Familiarity with medicine was evaluated through the ratio between the number of well-identified medicines to the total number of medicine of the patient.

If agreed to by the patient, the interview was performed at the patient’s home. In that case, data were also collected on the patient’s medicine cabinet (location and size) and the latter’s contents (the number of out-of-date medications, redundant medications, the presence of several generic formulations of a drug with the same dose level, and the presence of potentially inappropriate and/or at-risk medications with regard to the patient’s comorbidities and other treatments). Potential inappropriateness was assessed on the basis of both implicit and explicit criteria [18]. The questionnaire for medicine cabinet inspection is provided in Additional file 2.

### Statistical analysis

Quantitative variables were quoted as the mean ± standard deviation (SD) or (for non-normal distributions) the median [interquartile range (IQR)]. Qualitative variables were quoted as the number (percentage). Normal distribution was checked by graphical method (histogram and density curves).

One of the study’s objectives was to identify factors associated with difficulties in preparing and taking medications. The dependent variable was “difficulty in preparing or taking medications”, which corresponded to the aggregate replies to three questions in the study questionnaire (on difficulties in preparing, taking and/or swallowing medications). Firstly, a logistic bivariate regression was used to probe associations between the dependent variable and the other descriptive variables from the questionnaire. Variable with more than 10% of missing data were excluded from the analysis. This model generated a log-linear relationship between the dependent variable and the continuous variables. The log-linearity hypothesis was checked in a cubic spline approach, and was found to hold for the “age” variable (which was then fed into the model as a continuous variable).

Secondly, a multivariate logistic regression model was applied. Given that this was a pilot study with no prior knowledge of which variables should be selected first, all dependent variables with a *p*-value below 0.20 in the bivariate analysis were included in the multivariate analysis. The variables were then selected in a forward and then backward step-wise analysis. Multi-collinearity was checked by the measure of the variance inflation factor and The goodness-of-fit was assessed by the Hosmer-Lemeshow test.

All statistical analyses were performed with R software (version 3.2.0) [19].

## Results

### Characteristics of the study population

The 141 students performed interviews with a total of 1370 patients, including 743 (54.2%) who agreed to an assessment of the home medicine cabinet. The characteristics of the patients and their medications are summarized in Table 1. The mean ± SD age was 81.5 ± 5.7. Nearly one in two of the patients (48.5%) lived on their own, and 43.8% of the patients had a home help. Over half of the patient reported suffering from geriatric syndromes: 51.5% of the patients had already had a fall, 66.1% had problems walking, and 23.8% had lost weight in the previous 6 months. Moreover, 23.7% of the patients had been hospitalized in the previous 6 months.

**Table 1:** Characteristics of the study population

	MD (%)	Patients (*n* = 1370)
Age, years [mean ± SD]	0.6	81.5 ± 5 .7
Living alone at home [n(%)]	0.9	659 (48.5%)
Home help [n(%)]	1.5	592 (43.8%)
Falls (≥ 1 in the previous 12 months) [n(%)]	3.0	685 (51.5%)
Difficulty walking [n(%)]	45.7	492 (66.1%)
Recent weight loss [n(%)]	2.2	319 (23.8%)
Hospitalization in the previous 6 months [n(%)]	1.2	321 (23.7%)
Self-reported medical history	3.1	
Dementia [n(%)]		74 (5.6%)
Dyslipidemia [n(%)]		652 (49.1%)
Diabetes [n(%)]		445 (33.5%)
Heart failure [n(%)]		320 (24.1%)
Myocardial infarction [n(%)]		186 (14.0%)
LEAOD [n(%)]		108 (8.1%)
Chronic kidney failure [n(%)]		74 (5.6%)
Other [n(%)]		1034 (77.9%)
Number of medications taken daily [mean ± SD]	11.5	9.3 ± 3.2
Number of OTC medications taken daily [median (IQR)]	28.6	0 [0; 1]
Self-reported adverse events [n(%)]	1.7	566 (42.0%)
Self-medication [n(%)]	1.6	426 (31.6%)
Knowledge of what the medications are for	2.6	1084 (81.3%)
Familiarity with medications	11.9	
Familiarity with < 25% [n(%)		108 (8.9%)
Familiarity with 25–50% [n(%)]		102 (8.5%)
Familiarity with 50–75% [n(%)]		187 (15.5%)
Familiarity with ≥ 75% [n(%)]		810 (67.1%)
French health insurance adherence questionnaire	2.0	
Good adherence [n(%)]		608 (45.3%)
Minor adherence problems [n(%)]		616 (45.9%)
Poor adherence [n(%)]		118 (8.8%)
Difficulties in preparing or administering medications [n(%)]	0	378 (27.6%)
Difficulties in preparing [n(%)]	4.2	164 (12.5%)
Difficulties in administering [n(%)]	3.3	130 (9.8%)
Difficulties in swallowing [n(%)]	1.2	154 (11.4%)

*Abbreviations*: *MD* Missing data, *SD* Standard deviation, *LEAOD* Lower extremity arterial occlusive disease, *OTC* Over-the-counter

### Treatment adherence, and knowledge about medications

The mean ± SD number of medications taken daily was 9.3 ± 3.2. Self-medication was reported by 340 patients (34.8%; missing data = 392) taking a median [IQR] of 1 [1;2] over-the-counter medications per day (maximum: 15). Adverse events were reported by 566 patients (42.0%).

Most of the patients (81.3%) said that they knew which medications they were taking. This statement was corroborated by the fact that the indication for at least 75% of their daily medicines was known to 61.7% of the patients. On the questionnaire recommended by the French national health insurance, 45.3% of the patients displayed good adherence, and 8.8% displayed poor adherence. The remaining patients presented minor adherence problems; many of the patients felt that they had too many pills to take each day, and therefore sometimes stopped taking their medication (*n* = 434 (32.6%) replied “yes” to question 6 on the questionnaire recommended by the French national health insurance).

When preparing their medications, 64.2% of the patients referred to their prescription, and the remainder referred to the information written on the medication’s packaging by the pharmacist. About one in two patients (53.5%) owned a pill box. In total, 261 (19.1%) interviews led to a discussion with the patient’s family physician about particular points noted by the pharmacy student.

### Patients encountering difficulties

After the fulfilment of their prescriptions at the community pharmacy, more than a quarter of the patients (*n* = 378; 27.6%) reported difficulties at home with regard to medication preparation, administration and/or swallowing (Table 1). The results of the bivariate and multivariate analyses are summarized in Table 2. Variance inflation factor was lower than 1.5 for all covariates, suggesting the absence of multi-collinearity; the goodness-of-fit was satisfactory according to the Hosmer-Lemeshow test (*P* = 0.6328). The multivariate analysis showed that older age, the presence of a home help, self-reported adherence problems, and the occurrence of adverse events were independently associated with a greater likelihood of difficulties in preparing and/or taking medication at home. In contrast, the lack of third party assistance when taking medication was a protective factor.

**Table 2:** Bivariate and multivariate analysis of the factors associated with difficulty preparing or administering medications at home

	Bivariate analysis		Multivariate analysis	
	OR	95% CI	OR	95% CI
Age	1.04	1.02–1.06	1.03	1.00–1.05
Living alone	1.02	0.79–1.31		
Home help	1.98	1.53–2.56	1.59	1.20–2.09
Dementia	1.80	1.06–3.08		
Falls	1.20	0.93–1.55		
Recent weight loss	1.45	1.09–1.92	1.25	0.93–1.69
Hospitalization in the previous 6 months	1.32	0.99–1.75		
Self-reported adverse events	1.61	1.25–2.08	1.50	1.15–1.96
Medications prepared by the patient	0.60	0.44–0.80		
Medications prepared with reference to the prescription	0.90	0.69–1.17		
Medications prepared with reference to the information written on the box by the pharmacist	1.13	0.88–1.46		
Use of a pill box	1.24	0.96–1.60		
Medications administered by the patient	0.41	0.28–0.61	0.53	0.35–0.80
Knowledge of the treatment	0.58	0.43–0.78	0.77	0.55–1.06
French health insurance adherence questionnaire				
Good adherence	1		1	
Minor adherence problems			1.33	1.01–1.77
Poor adherence	3.15	2.04–4.86	2.63	1.67–4.14

*Abbreviations*: *OR* Odds ratio, *CI* Confidence interval

### Medicine cabinet inspections

The pharmacy students performed 743 inspections of the patient’s home medicine cabinet (Table 3). On average, the visit lasted 14 ± 10.2 min. In the majority of cases (76.5%) there was only one home medicine cabinet. The medicine cabinets were variously located in the kitchen, living room, bathroom, and bedroom. In 15.0% of cases, the medicine cabinet was considered to be in an unsuitable location for various reasons, such as a room with high levels of humidity or a hard-to-reach place. More than a third of the patients shared their medicine cabinet: 30.4% shared with their spouse, and 6.0% shared with another person (usually their son or daughter).

**Table 3:** Characteristics and content of the home medicine cabinets inspected (*n* = 743)

	MD (%)	Cabinets inspected (*n* = 743)
A single home medicine cabinet	0.5	565 (76.5%)
Unsuitable location	6.6	104 (15.0%)
Location:	26.0	
Kitchen		192 (34.9%)
Living room		146 (25.6%)
Bathroom		109 (19.8%)
Bedroom		54 (9.8%)
Other room		49 (8.9%)
Type of medicine cabinet:	0.4	
Cupboard		406 (54.9%)
Bag		61 (8.2%)
Drawer		172 (23.2%)
Other		171 (23.1%)
Medicine cabinet used by:	3.1	
The patient only		510 (69.3%)
The patient and his/her spouse		224 (30.4%)
The patient and another person		44 (6.0%)
Storage problems	5.8	152 (21.7%)
Number of expired drugs:	1.9	
0		432 (59.3%)
1–5		201 (27.6%)
6–10		48 (6.6%)
11–15		19 (2.6%)
≥ 16		29 (4.0%)
Storage of products other than drugs	1.2	181 (24.7%)
Presence of potentially inappropriate and/or at-risk medications	6.6	104 (15.0%)
Redundant medications	3.1	147 (20.4%)
Different generic formulations of the same drug at the same dose level	2.8	137 (19.0%)
Patient with medications kept in the refrigerator	0.8	129 (17.5%)
Inappropriate storage in the refrigerator (*n* = 129)	0.8	22 (3.0%)

*Abbreviation*: *MD* Missing data

In 152 cases (21.7%), storage problems were noted. In most cases, these were related to medications lacking their packaging and information sheets. Expired medications were found in 40.7% of the medicine cabinets, and over 25% of the cabinets contained three or more expired medications. One medicine cabinet contain 66 expired medications, and other contained a medication that had expired in 1992 (23 years previously). Information about the regular use or not of expired medication was not collected. Furthermore, 24.7% of the patients were storing other products (e.g. veterinary medications, cosmetics, hygiene products and even foodstuffs) with their medications.

One or more potentially inappropriate and/or at-risk medications (with regard to a patient’s comorbidities and treatments) were identified in 15.0% of the medicine cabinets. Several different generic formulations of a given drug at the same dose level were found in 19.0% of the medicine cabinets inspected. Moreover, redundant medications were evidenced in 20.4% of the medicine cabinets.

## Discussion

The present study provided a particularly valuable description of older patients attending community pharmacies in the north of France, and their home medications. Our results revealed that more than a quarter of the patients had difficulty preparing or administering medications at home. A total of over 700 medicine cabinet inspections identified some problems related to poor location/storage and the frequent presence of inappropriate and redundant medications. Our identification of difficulties in home medication management opens up new opportunities for caring for older outpatients; the community pharmacist is particularly well placed to address these difficulties.

The patients included in the present study were representative of older people attending community pharmacies. On average, a patient was taking 9.3 prescription medications a day. This high number is similar to that found in a general-population survey of over 200,000 people living in the region of France where the present study was performed [20]. Furthermore, many of the older people in our study reported geriatric syndromes, such as falls, balance disorders and weight loss. These findings are suggestive of a high proportion of frail patients (estimated at 39–45% in people aged 85 and over [21]).

The patients had a satisfactory level knowledge of their medications, and 67% of the patients were familiar with at least 75% of their medications. These findings are similar to those recorded in other European countries [22, 23]. A comparison of two studies performed in Denmark and Sweden suggested that knowledge of medications increased over time, since the proportion of patients familiar with at least 75% of their medications rose from 60% in 2000 to 71% in 2009 [22, 23]. Our study further showed that the older people overestimate their level of knowledge because 81.3% thought that they were familiar with all their medications. Barat et al.’s study also showed that only 4% of the 348 included older patients had been informed about the risk of adverse events [22]. In a Finnish study of older adults, only 11.4% of the 404 interviewees reported an adverse event [24]. Self-reported adverse events were more frequent in our study; 42% of the interviewees reported one or more event. However, the participants in the Danish and Finnish studies were taking fewer medications than the participants in our study, and a high number of medications is a major risk factor for adverse drug reactions [25, 26]. Several studies have highlighted the potential value of patient-led pharmacovigilance reporting of adverse events, as a complement to reporting by physicians [27, 28]. Our results suggest that there is significant potential for improving pharmacovigilance reporting by older patients, and that community pharmacists could usefully contribute to this process.

One important finding in the present study was that over a quarter of older patients had difficulty taking their medications at home, i.e. after the physician has issued a prescription and the pharmacist had provided the medication and corresponding advice. Although difficulties encountered by older people when taking medication have rarely been studied, these problems have been linked to poor adherence [29–31] - as also found in the present study. Our multivariate analysis identified several other factors associated with these difficulties, such as the need for a home help or the self-reported adverse events. However, not all of these factors can be easily spotted in the community pharmacy. Our results suggest that asking older people a few simple questions is enough to identify patients with difficulties. Discussing medications with a patient and his/her family might enable the pharmacist to suggest appropriate solutions (such as the implementation of a medication schedule, changes in pharmaceutical formulations, or the use of a pill box, tablet cutter or an eye dropper bottle) or to discuss the possible value of home assistance with medication administration. Multidose drug dispensing can also help the patients - notably with regard to better treatment adherence [32].

Another strength of our study relate to the 743 home visits with an inspection of the medicine cabinet. Medication storage problems were very common; they varied from an inappropriate location to the presence of redundant, inappropriate and/or at-risk medications. Many of these problems could be resolved by a few simple recommendations on home medication management from the pharmacy students. Our present findings appear to be of value because there are few published data on older people’s home medicine cabinets [33]. In a study of 86 older patients in Hong Kong, Lee et al. found that (i) 69.7% of the participants had at least one medication storage problem, and (ii) these problems were strongly correlated with poor adherence (odds ratio 10.3 (95% confidence interval: 2.5–44.6); *P* < 0.001) [33]. In Lee et al.’s study, intervention by a pharmacist often resolved the patient’s storage problems [33].

Lastly, our results showed that it is possible to raise awareness and train tomorrow’s pharmacists in these new roles via a structured, innovative adaptation of the mandatory, final-year internship in a community pharmacy.

Our study had a number of strengths, including the large sample size, the high proportion of frail patients, the use of standardized questionnaires, the large number of home medicine cabinets inspected, and performance of interviews by pharmacy students (who proved themselves to be well qualified for addressing these topics).

The study also had some limitations. The elderly participants were selected by the community pharmacist supervising the internship, and half of the patients were not available or refused an inspection of their home medicine cabinet. Our results might therefore reflect the characteristics of people who had a good opinion of healthcare professionals, and so the significance of some characteristics may have been over- or underestimated. Geriatric syndromes were assessed by simple questions and were not evaluated by dedicated tools. Prevalence of geriatric syndromes may therefore have been under- or over-estimated. Information about the regular use or not of expired medication was not available, nor if expired medications concerned regularly used medications. Consequently, the potential danger of expired medications stored in the medicine cabinets could not be estimated. Furthermore, the “difficulty in taking medication” parameter was determined on a post-hoc basis from prospectively collected data. However, difficulties in taking medication have rarely been studied, and the scale of the problem could not be anticipated when the study questionnaire was drafted. Consequently, there was no sample size calculation and associations identified in the multivariable analysis may be due to unmeasured cofounding factors. Another limitation relates to the fact that the short study period prevented us from assessing any improvements in home medication management after an interview with the patient’s family physician (i.e. a discussion of points raised by the medication review). Lastly, the study was performed in a single region of France, and so our observations may be specific to the cultural setting and/or the French healthcare system.

## Conclusion

Community pharmacists are well placed to ask simple questions that identify high-priory patients for pharmaceutical intervention and a medication review. In a context of polypharmacy, the often frail older people attending community pharmacies frequently have difficulty in managing, storing, preparing and administering their medications.

## Supplementary information

**Additional file 1.** Topic guide for the management of personal treatment and side-effects.

**Additional file 2.** Topic guide for the medicine cabinet inspection.

## Data Availability

The data supporting the findings can be obtained on reasonable request to the corresponding author.

## Abbreviations

IQR: Interquartile range
SD: Standard deviation
