# Supplementary material for: Polypharmacy in older patients: identifying the need for support by a community pharmacist
Source: BMC Geriatr. 2019 Oct 21;19:277. doi: 10.1186/s12877-019-1276-y (PMC6802481; doi:10.1186/s12877-019-1276-y)
Supplement: Supplementary file 1 — Additional file 1. Topic guide for the management of personal treatment and side-effects. [file 12877_2019_1276_MOESM1_ESM.pdf]

Topic guide - Management of personal treatment & iatrogeny

Patient identification (to be prepared before the interview)

Name, first name: Age:

Name (telephone number) of the attending physician:

Weight: kg Size: m

Postal code of residence:

Open PD: ☐ Yes ☐ No (*if no, try to convince the patient to open a PD*)

Hospital discharge: ☐ Yes ☐ No

Comorbidities

☐ Memory Disease ☐ Myocardial Infarction

☐ Dyslipidemia ☐ peripheral arterial occlusive disease

☐ Diabetes ☐ Chronic renal failure

☐ Heart failure ☐ Other:

Current treatment (number of prescribed drugs: + self-medication:)

Indicate the drugs (specialty name, dosage and international non-proprietary name):

- -
- -
- -
- -
- -
- -
- -
- -

Points of attention regarding treatment: ☐ Yes ☐ No

Which ones:

Date of the first interview:

Suggest to the patient to come with his biological assessments

Dates of the following dispensations:

Interview with the patient ☐ with the caregiver ☐

Treatment & Iatrogeny

Do you know what your medications are used for? ☐Yes ☐No
Number of drugs identified: / total number of drugs :

Do you know what precautions to take with your treatments? ☐Yes ☐No
Target, if applicable, KIAs, hypoglycemic agents, CKD management, pain medications

Did you know that some of your medications can disrupt driving?
☐Yes ☐No

In case of "banal" pain or low fever, do you know the most appropriate medication?
☐Yes ☐No

Do you ever take medication on your own initiative? (including with some food supplements or plants) ☐Yes ☐No

Do you ever change the dosage of drugs on your own initiative?
☐Yes ☐No

What do you do if you forget to take one of your medications?

Do you get vaccinated regularly? ☐Yes ☐No

Have you ever had any adverse reactions or unpleasant reactions with your treatment?
☐Yes ☐No

Can you describe them? What is your attitude in this case?

Have you ever fallen? ☐Yes ☐No
How many times and when?
Under what conditions?
Do you have difficulty walking?

Have you lost weight recently? ☐Yes ☐No

Processing management

Have you been hospitalized in the last 6 months? ☐ Yes ☐ No
If so, why not?

Do you live alone? ☐ Yes ☐ No

Do you have a home helper? ☐ Yes ☐ No

Who prepares your treatment? ☐ You yourself ☐ Your spouse ☐ Other:

How? ☐ from the prescription ☐from information written by your pharmacist on the boxes?

Do you have difficulties preparing your treatment? ☐ Yes ☐ No
Which ones?
Evaluate possible reading difficulties and/or vision/dexterity problems that could have an impact on treatment follow-up

Do you use a pill dispenser? ☐ Yes ☐ No
Possibly propose one that the association of tutors has provided you with
If so, can you show me how you use it?

Do you take your treatment alone/independently? ☐ Yes ☐ No

Do you have a stock of medicines in your home? ☐ Yes ☐ No
Can I come and see them to sort it out? ☐ Yes ☐ No

Do you have difficulty swallowing medications? ☐ Yes ☐ No
If so, how do you do it?
Identify potential problem drugs.
If so, how many?

Do you have any difficulties taking certain medications?
☐ Yes ☐ No
Ask concretely how they do it (eye drops, ½ tablets, inhaler...)

TIME SPENT FOR THE INTERVIEW:

SUMMARY OF THE POINTS OF REFERENCE DURING THIS INTERVIEW

In case of attention points, formulate the elements of the interview with the attending physician to be validated by your internship supervisor before contact with the attending physician.

REPORT OF THE CONTACT WITH THE DOCTOR

Specify the consequences of this interview for the patient's management.
