# Supplementary material for: Polypharmacy in older patients: identifying the need for support by a community pharmacist
Source: BMC Geriatr. 2019 Oct 21;19:277. doi: 10.1186/s12877-019-1276-y (PMC6802481; doi:10.1186/s12877-019-1276-y)
Supplement: Supplementary file 2 — Additional file 2. Topic guide for the medicine cabinet inspection. [file 12877_2019_1276_MOESM2_ESM.pdf]

# Topic guide for the medicine cabinet inspection

**Key messages to deliver**

- Keep the medicines in their original packaging and with their package leaflet
- Sort out the expired or unused drugs regularly and have the reflex CYCLAMED by reporting them to your pharmacy
- Do not keep any medications (unless otherwise stated) such as antibiotics, or those that quickly perish after opening (syrups, drops, eye drops, nasal solutions)
- Separate your treatment from those around you
- Do not reuse a drug that has been previously prescribed without the advice of your pharmacist or doctor
- Keep your medicine in one place of the house and out of reach of children

**HOUSING**

Take advantage of the visit to appreciate the risks of falling in the home, for example, the presence of carpets, unevenness, small steps and, if necessary, give advice at this level to prevent risks. Watch the patient's footwear and give advice if needed.

**If you have proposed an adaptation, please specify.**

**Medicine cabinet**

Is this the only home medicine cabinet? ☐ Yes ☐ No

Where it is?

Is the place suitable? ☐ Yes ☐ No

Why?

What is the format of the medicine cabinet?

☐ A cupboard
☐ A bag
☐ One drawer
☐ Other:

Who uses this medicine cabinet?

☐ The elderly person
☐ The couple
☐ Other people

*If it is a cabinet used by several people, identify the treatments of each person*

Are medicines stored in the refrigerator? ☐ Yes ☐ No
If Yes, is the place of storage in the refrigerator suitable? ☐ Yes ☐ No
*In case of a negative answer, re-specify the storage rules.*

**The content of the cabinet**

Are other types of products stored with the drugs? ☐ Yes ☐ No
If yes, specify

Have you identified potentially inappropriate and / or at-risk drugs (ie, completed antibiotic treatment, non-prescribed anti-inflammatory drug)? ☐ Yes ☐ No
*In case of clearly identified problem and after discussion with the patient, remove the relevant medications from the cabinet. Inform possibly the prescriber.*
If Yes, specify the nature of the problem and the number of specialties involved:

Have you identified any expired medicines? ☐ Yes ☐ No
*Remove the medications concerned from the cabinet.*
If Yes, specify the number of boxes:

Have you identified any expired medications within 6 months? ☐ Yes ☐ No
*Discuss with the patient about a possible recovery. Suggest to indicate in a very visible way the expiry date on the box.*
If Yes, specify the number of boxes:

Have you identified any storage problems (ie, opened vials, wafer without packaging ...)?
☐ Yes ☐ No
*Remove the medications concerned from the cabinet.*
If Yes, specify the nature of the problem and the number of boxes:

Have you identified therapeutic redundancies? ☐ Yes ☐ No
If Yes, specify the specialties:

Have you identified different generics of the same proprietary medicinal product at the same dosage?
☐ Yes ☐ No

Are they correctly identified and recognized by the older patient? ☐ Yes ☐ No
Are they correctly identified and recognized by the caregiver? ☐ Yes ☐ No

Have you identified specialties whose active ingredient (s) are identical but at different dosages?
☐ Yes ☐ No

Time spent when visiting the cabinet:
